# A bibliometric and visual analysis of cognitive function in bipolar disorder from 2012 to 2022

**DOI:** 10.1186/s12991-024-00498-x

**Published:** 2024-04-18

**Authors:** Xiaohong Cui, Tailian Xue, Zhiyong Zhang, Hong Yang, Yan Ren

**Affiliations:** 1 Department of Psychology, School of Humanities and Social Sciences, Shanxi Medical University, Taiyuan, Shanxi China; 2 Shanxi Bethune Hospital, Shanxi Academy of Medical Sciences, Tongji Shanxi Hospital, Taiyuan, 030032 China; 3 Department of Psychiatry, Third Hospital of Shanxi Medical University, Shanxi Bethune Hospital, Shanxi Academy of Medical Sciences,Tongji Shanxi Hospital, Taiyuan, 030032 China; 4 Shanxi Bethune Hospital, Shanxi Academy of Medical Sciences, Tongji Shanxi Hospital, Third Hospital of Shanxi Medical University, Taiyuan, 030032 China; 5 Tongji Hospital, Tongji Medical College, Huazhong University of Science and Technology, Wuhan, 430030 China

**Keywords:** Bipolar disorder, Cognitive function, Bibliometric, Visualize

## Abstract

**Introduction:**

Bipolar disorder (BD) is a chronic psychiatric disorder that combines hypomania or mania and depression. The study aims to investigate the research areas associated with cognitive function in bipolar disorder and identify current research hotspots and frontier areas in this field.

**Methodology:**

Publications related to cognitive function in BD from 2012 to 2022 were searched on the Web of Science Core Collection (WoSCC) database. VOSviewer, CiteSpace, and Scimago Graphica were used to conduct this bibliometric analysis.

**Results:**

A total of 989 articles on cognitive function in BD were included in this review. These articles were mainly from the United States, China, Canada, Spain and the United Kingdom. Our results showed that the journal “*Journal of Affective Disorders*” published the most articles. Apart from “Biploar disorder” and “cognitive function”, the terms “Schizophrenia”, “Meta analysis”, “Rating scale” were also the most frequently used keywords. The research on cognitive function in bipolar disorder primarily focused on the following aspects: subgroup, individual, validation and pathophysiology.

**Conclusions:**

The current concerns and hotspots in the filed are: “neurocognitive impairment”, “subgroup”, “1st degree relative”, “mania”, “individual” and “validation”. Future research is likely to focus on the following four themes: “Studies of the bipolar disorder and cognitive subgroups”, “intra-individual variability”, “Validation of cognitive function tool” and “Combined with pathology or other fields”.

**Supplementary Information:**

The online version contains supplementary material available at 10.1186/s12991-024-00498-x.

## Introduction

Bipolar disorder presents a complex clinical presentation. It is characterized by alternating episodes of mania and depression, and is a serious mental health problem with a high rate of disability and difficult to cure [1]. The phenotypic manifestations of BD include not only core abnormalities in mood regulation, but also cognitive impairments, sleep/wake disturbances, and a high prevalence of comorbidities in both internal medicine and psychiatry. Cognitive function, also known as neurocognitive function, refers to the ability of the human brain to process information, including memory, executive function, space, time, language comprehension and expression. The current cognitive dysfunction in patients with bipolar disorder primarily affects memory, attention, executive function and so on [2].

In recent years, there has been increasing attention on cognitive functioning in patients with bipolar disorder. For example, a study has shown that individuals with bipolar disorder experience cognitive impairments during periods of remission as well as during acute episodes of depression or mania. Cognitive impairment is associated with multiple factors, including age of onset, duration of remission, and cognitive impairment, which are also intrinsic phenotypes of the disease [3]. Different diseases cause varying degrees of cognitive impairment. Patients with schizophrenia exhibit comprehensive cognitive decline, while those with bipolar disorder primarily experience impairment in memory, attention, and executive function, especially during acute episodes. The classification of bipolar disorder also corresponds to different areas of cognitive impairment. The impact of medication treatment on the cognitive function of bipolar disorder patients is contradictory, requiring a combined approach with other therapeutic methods to improve patient cognition [4]. The assessment of cognitive function is also a research prominent topic in this field. The assessment of cognitive function in bipolar disorder includes both objective and subjective aspects. Various neuropsychological tests, such as the Rey Auditory Verbal Learning Test (RAVLT) and Rapid Visual Information Processing (RVP) test, are used to assess objective cognitive function in individuals with bipolar disorder. Subjective cognitive function assessment can be performed using the Cognitive and Physical Functioning Questionnaire (CPFQ) [5]. In addition to the above assessment tools, the results of another study showed: for patients with BD in partial or full remission, the Screen for Cognitive Impairment in Psychiatry (SCIP) and the Cognitive Complaints in Bipolar Disorder Rating Scale (COBRA) are effective tools for screening objective and subjective cognitive impairments, respectively [6]. These findings indicate that we need to assess different aspects of cognitive impairment in patients using various scales. This will help us better understand their cognitive performance and provide assistance for clinical treatment.

Bibliometrics is the analysis of published information (books, journal articles, datasets, blogs) and associated metadata (abstracts, keywords, citations). It describes or shows the relationship between published works by using statistical data [7]. The characteristics of publications and the relationships between publications can be described by qualitative and quantitative analysis. Cognitive function is currently a hot topic of research in bipolar disorder, but there are no bibliometric articles yet, although there are many articles on cognitive function in bipolar disorder.

In this article, we use CiteSpace and VOSviewer software to review the research of cognitive function in bipolar disorder in the past 12 years (from 2012 to 2022) to learn the status of international research, the shift in research hotspots and emerging trends in this field.

## Research methodology

### Data sources and search strategy

**Fig. 1:**
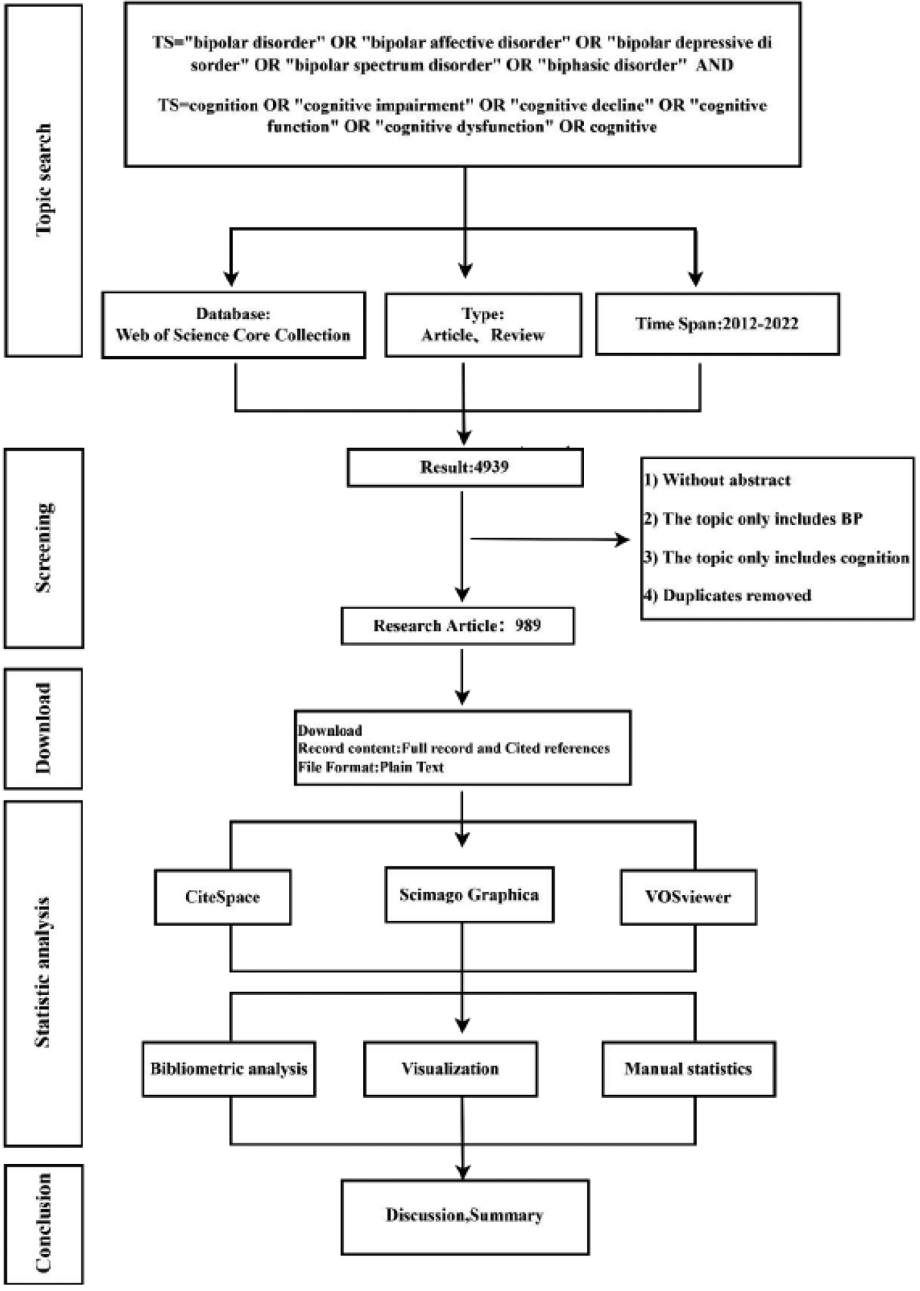
Flow chart of scientometric analysis

This study searched the related literature of cognitive function in bipolar disorder from the Web of Science (core collection) database, The reason for choosing WoSCC is that it is a high-quality digital literature resources database, suitable for the quantitative analysis of literature. The retrieval formula is TS="bipolar disorder” OR “bipolar affective disorder” OR “bipolar depressive disorder” OR “bipolar spectrum disorder” OR “biphasic disorder” AND TS = cognition OR “cognitive impairment” OR “cognitive decline” OR “cognitive function” OR “cognitive dysfunction” OR cognitive, selected over a period of 2012–2022, analyzed the type of literature as articles and reviews, and included the studies without regard to language. Through the analysis of the titles, abstracts and keywords of the article, a total of 4939 articles were searched, 1005 articles were preliminarily screened out, 989 articles were obtained (865 articles, 124 reviews) (Fig. 1).

### Research tools

In this study, VOSviewer software (version 1.6.18) on WoSCC database is applied to conduct co-occurrence analysis, combined with Scimago Graphica software to achieve a country map visualization analysis. Using CiteSpace (version of 6.1.R6) software for the database author co-operation analysis, keyword cluster analysis, literature co-citations and keyword mutation analysis. VOSviewer application developed by Nees Jan van Eck and Ludo Waltman (Leiden University) in 2010, can be used for a variety of network analysis, including collaborative analysis, keyword co-occurrence analysis, citation and co-citation analysis, and bibliographic coupling. It can be used to conduct co-authorship analysis, keyword co-occurrence analysis, citation and co-citation analysis, and bibliographic coupling [8]. Citespace is a software developed by Professor Chaomei Chen of Drexel University (Philadelphia, USA) for the visual analysis of scientific references. With the software, we can generate a series of visual knowledge atlases to understand the research hotspots in the field, delve into the forefront of its development, and ascertain emerging trends [9].

The parameters used for co-occurrence analysis using VOSviewer are the default parameters for the software, and the parameters used in CiteSpace are as follows: time slices (2012–2022), number of years per slice (1), node types (author collaborations, co-citations, and keywords), pruning (pathfinder, pruning sliced network, pruning the merged network), g-index (k = 25, literature co-citations are k = 15).

## Results

### Analysis of publication years

**Fig. 2:**
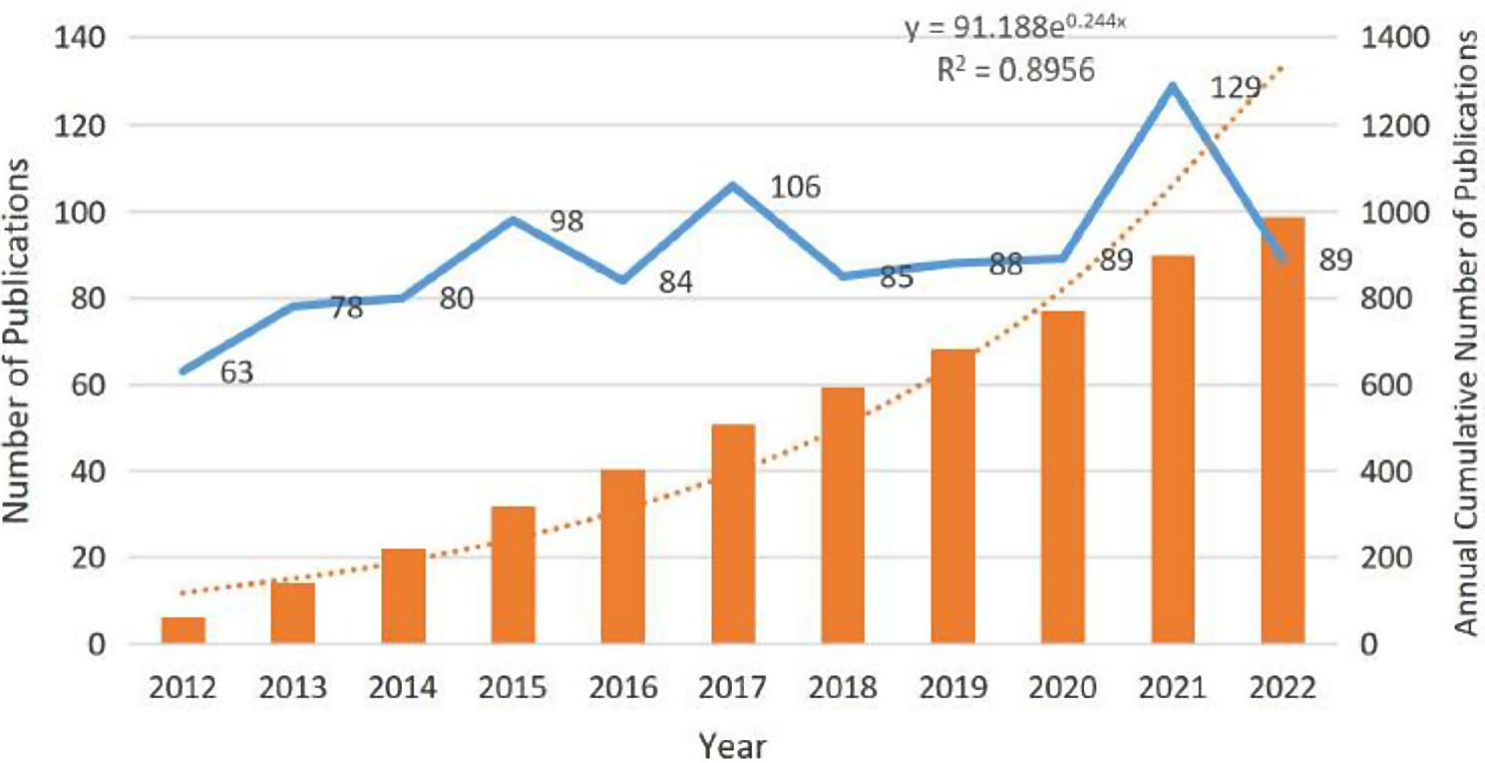
Distribution of publication from 2012 to 2022

There are a total of 989 articles were used in this study from 57 countries. Figure 2 shows the distribution of publication years for articles in the field of cognitive function in bipolar disorder. Overall, the volume of articles in this field is relatively balanced, with more than 60 articles published annually starting from 2012. The number of articles published in 2018–2020 remained relatively stable. The highest volume of articles was in 2021, with 129 articles published. The annual cumulative volume model aligns with the annual growth data y = 91.188e^0.244x^ (R ^2^ = 0.8956). This also shows that the field of cognitive function in bipolar disorder has garnered significant attention from scholars, with the development of society and technology, the study of cognition in the context of bipolar disorder has become an important and hot topic.

### Analysis of author

By analyzing the authors of the literature cited in this paper, we aimed to gain insights into the prominent scholars and core strength within this research area. Famous scholar Price pointed out that, in the same subject, half of the papers are written by a group of high-productivity authors, and the number of authors in this group is approximately equal to the square root of the total number of authors [10].

According to the Price’s Law, the minimum number of core authors in the field is m = 4.79, so authors with 5 or more posts (including 5) are positioned as core authors in the field, where they are active professionals. Table 1 shows the top five productivity authors with contributions in this area. Top of the list was Vieta E, professor and chair of psychiatry at the University of Barcelona, with the highest number of published articles (41). He spearheads research focused on investigating cognitive function, cognitive impairment, and clinical manifestations associated with bipolar disorder, leading the Bipolar Disorder and Depression Project in Barcelona, Catalonia, Spain.

**Table 1:** Top 5 most publication authors

Rank	Author	Documents	Centrality
1	Vieta, E	41	0.14
2	McIntyre, RS	23	0.07
3	Vinberg, M	23	0.02
4	Miskowiak, KW	20	0.09
5	Martine-Aran, Anabel	17	0.04

**Fig. 3:**
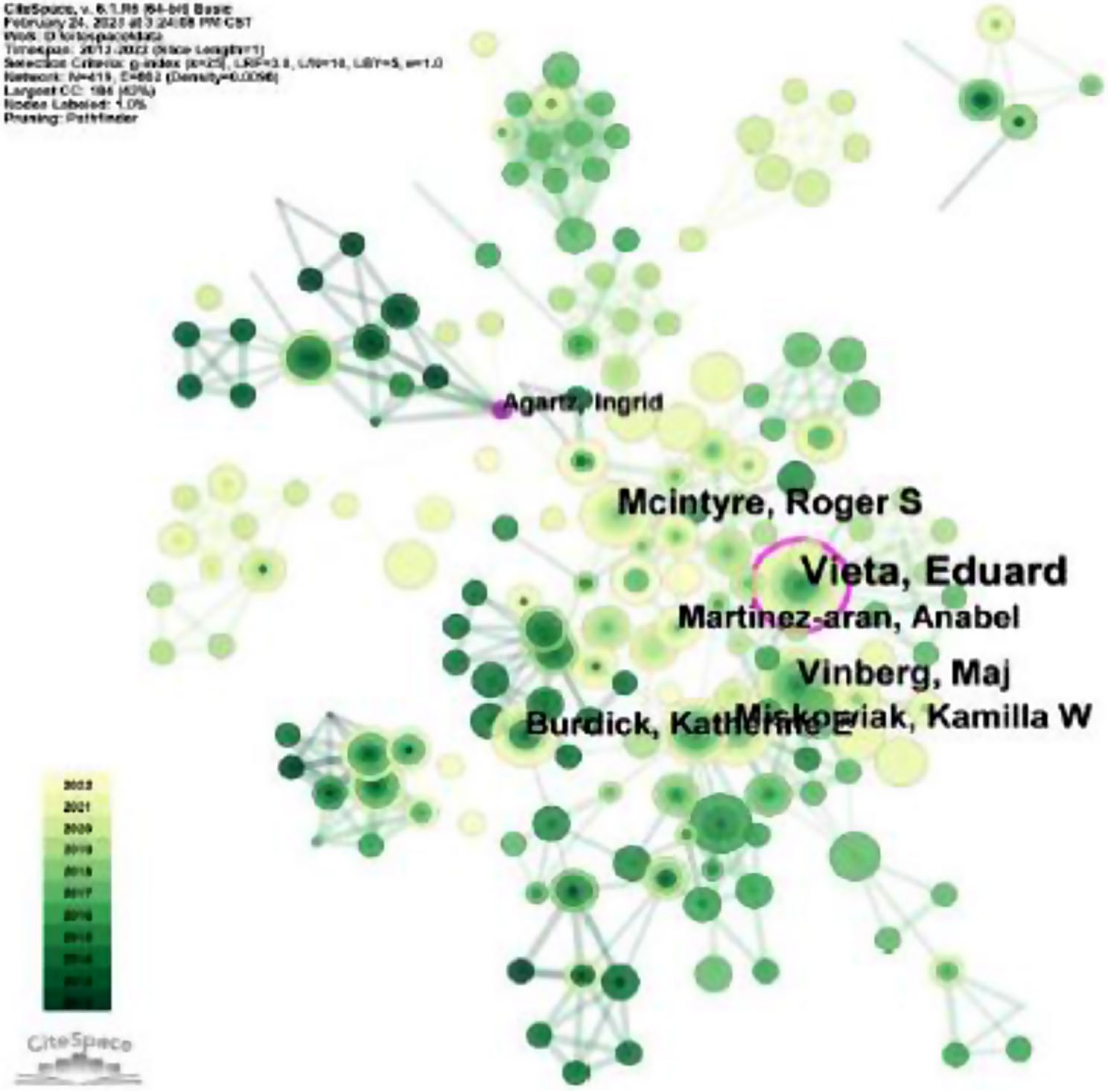
Author collaboration network analysis. The shorter the distance between two nodes the thicker the connection, indicating a higher level of collaboration between the two authors. Green nodes represent earlier published studies, while yellow nodes represent more recent studies

We then analyzed the author’s cooperation relationship. These studies published between 2012 and 2022, the year per slice for analysis is 1 year. The author cooperation network is shown in Fig. 3 (N = 419, E = 862). The circle size of the node represents the number of publications.

The centrality indicates that an author has a close cooperation relationship with other authors. According to Table 1, the centrality of Vieta E is 0.14 (centrality > 0.1), indicating that the author has cooperation with multiple authors in the field, while the centrality of Vinberg M is 0.02. The author’s cooperation network graph also shows that Vinberg M is far away from other high-yielding authors, indicating that the author has less cooperation with other high-yielding authors in the research of cognitive function in bipolar disorder.

### Analysis of the most productive journals

The analysis of the journals in literature shows that journals published in this field belong to the medical field except a few comprehensive journals in the past ten years. The top 10 most publication journals have shown in Table 2. Journals with more than 60 published articles were *Journal of Affective Disorders*, *Psychiatry Research* and *Bipolar Disorders*, with 185, 72, and 66 articles, respectively. Among them, *PLOS ONE* is an open-source journal, with 20 citations, ranking 10th in the number of published journals.

Analyzing of journal citation founds that (Table 2) the most cited journals are the top medical journal “*Acta Psychiatrica Scandinavica*”, with a total of 27 articles cited up to 47 times. It indicates that the journal publishes high-quality articles and is of widespread interest in the field of cognitive function in bipolar disorder. The contents published in this journal include: empirical studies, factor studies, and the influence of variable indicators on cognitive function in patients with bipolar disorder.

**Table 2:** Top 10 most publication journals

Rank	Source	Publications	Citations	Average Citation/Publication	IF(2022)
1	Journal of Affective Disorders	185	3807	20.58	6.533
2	Psychiatry Research	72	1150	15.97	11.225
3	Bipolar Disorders	66	2186	33.12	5.345
4	Psychological Medicine	34	848	24.94	10.592
5	Journal of Psychiatry Research	29	593	20.45	5.250
6	Acta Psychiatrica Scandinavica	27	1284	47.56	7.734
7	Frontiers in Psychiatry	26	192	7.38	5.435
8	Schizophrenia research	24	556	23.17	4.662
9	European Neuropsychopharmacology	19	596	31.37	5.415
10	PLOS ONE	14	283	20.21	3.752

**Fig. 4:**
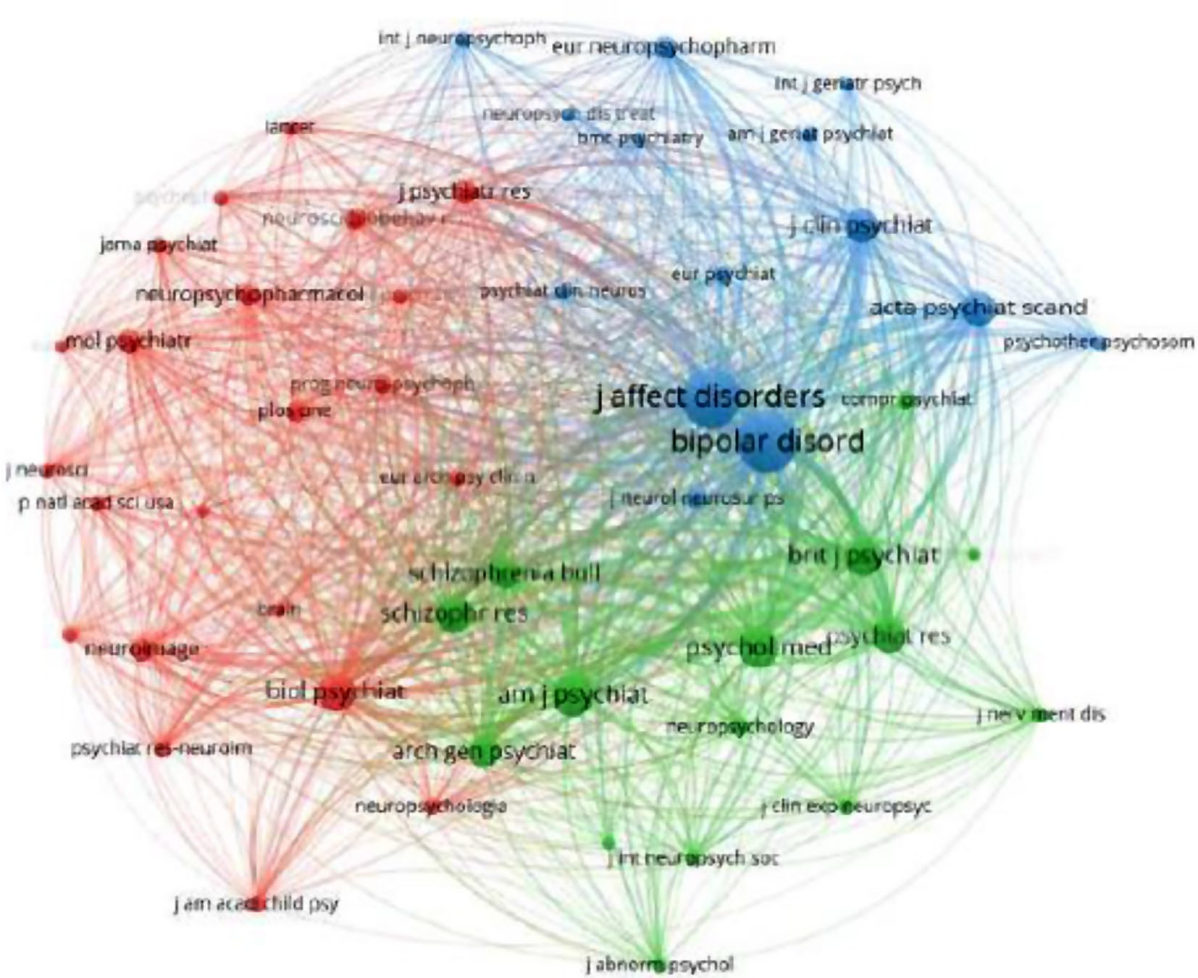
Co-citation resource

Furthermore, a visual analysis of the journal co-citation network reveals the presence of three clusters (Fig. 4, in supplementary material). According to the subject of the co-citation literature clustering, it divided into three different themes. The top 3 most cited journals are *Journal of Affective Disorders *(3807 citations), *Bipolar Disorders* (2186 citations), and *American Journal of Psychiatry* (1284 citations). All three of these journals are in the JCR1. The most cited journal, *Journal of Affective Disorders*, which includes articles on affective disorder. It covers a wide range of subjects, including neuroimaging, cognitive neuroscience, genetics, molecular biology, etc. This is in line with the research focus on cognitive function in bipolar disorder.

### Analysis of the most productive countries/regions

**Fig. 5:**
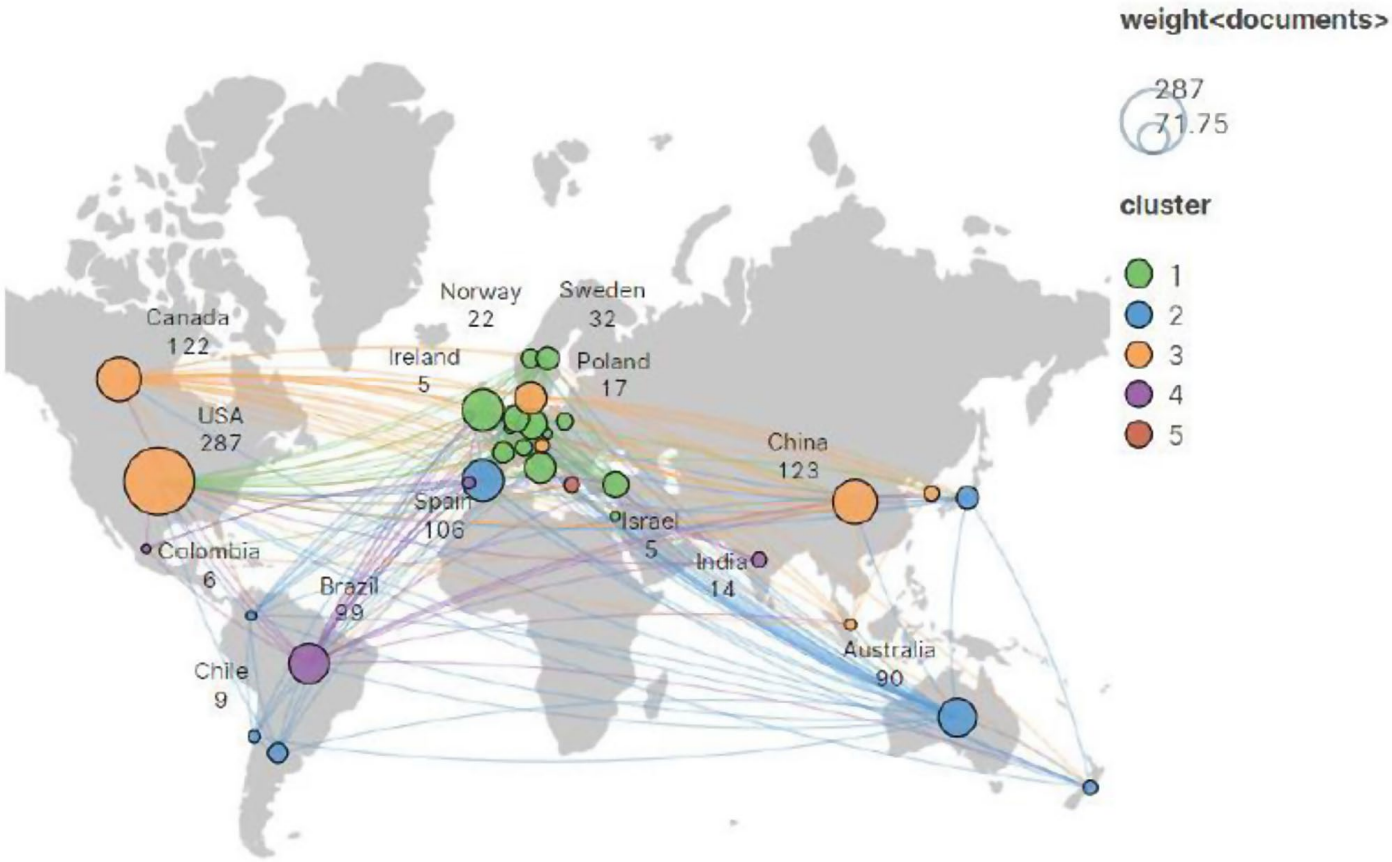
The visual map of countries. (**A**) The size of each node represents the number of publications from that country. (**B**) The number displayed below each country indicates the total number of publications

In 2012–2022, a total of 57 countries have published articles on cognitive function in bipolar disorder. To gain an understanding of the countries that have made significant contributions to this field, the study utilized VOSviewer to visualize the countries with 5 or more articles, a total of 33 countries met this criterion. The data was then exported in HTML format and imported into Scimago Graphica for visual analysis to generate map. The result is shown in the Fig. 5.

As we can see, most of articles are written by scholars from a few countries. Other countries such as Chile, Ireland, Colombia, etc. They have published fewer articles related to cognitive function in bipolar disorder in 2012–2022.

A further analysis of high-productivity countries in the field is presented in Table 3, showing the Top 5 countries in the field. According to the data in Table 3, American scholars have contributed the most research articles (287 articles published), accounting for 29% of the total number of articles published in this field. The second largest country is China, with 123 articles. The USA and the United Kingdom with a centrality value above 0.1, with centrality ratios of 0.25 and 0.36, respectively, indicating that these countries work closely with other countries in the study of cognitive function in bipolar disorder.

**Table 3:** Top 5 most publication countries

Rank	Country	Publications	Centrality
1	USA	287	0.25
2	China	123	0.08
3	Canada	122	0.08
4	Spain	105	0.05
5	United Kingdom	105	0.36

### Analysis of keywords

**Fig. 6:**
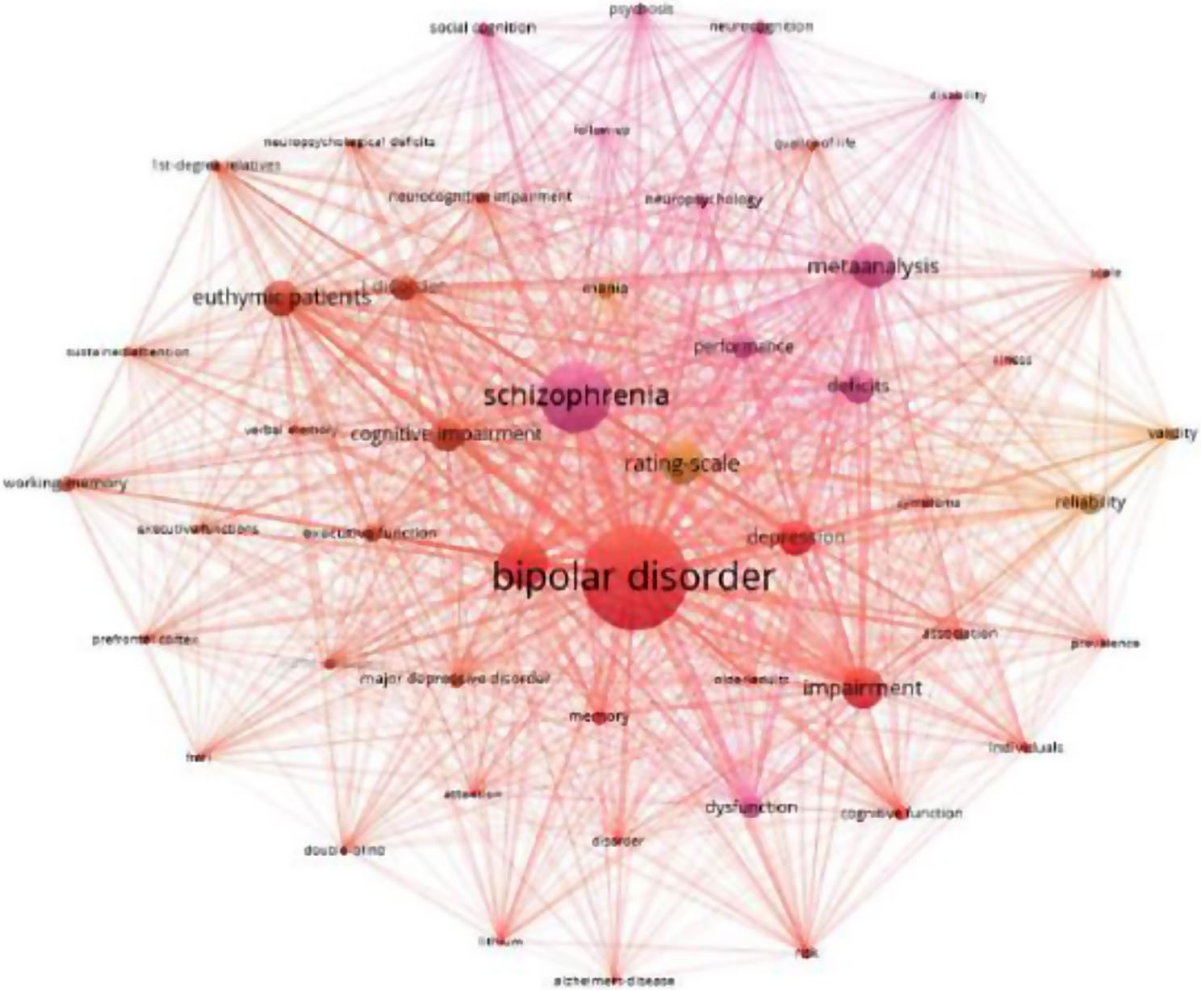
Co-occurrence analysis of keywords. (**A**) The size of the node represets the frequency of the keyword. (**B**) The distance between two node represets the strength of their association

We can classify keywords according to their frequency of occurrence and point out the links between high-frequency keywords. The analysis of keywords can help us understand the academic structure of a field and reveal the frontiers of research in the discipline. Figure 6 ( in supplementary material) presents the network and density of keywords that are referenced in the top 50. Keywords that are close to each other are classified into the same cluster, providing an overview of the main topics related to cognitive function in bipolar disorder.

In terms of co-occurrence frequency, the most frequent keyword was “Biploar disorder” with 712 citation times, followed by “Schizophrenia”, “Cognition”, “Meta analysis”, “Rating scale”, “Impairment”, “Euthymic patients”, “Deficits”, “Depression” and “Cognitive impairment” (Table 4). These high-frequency keywords reflect the hot spots of cognitive function in bipolar disorder.

**Table 4:** Top 10 keywords with the highest frequency of occurrence

Ranking	Keyword	Frequency	Ranking	Keyword	Frequency
1	Biploar disorder	712	11	I disorder	156
2	Schizophrenia	460	12	Performance	146
3	Cognition	308	13	Reliability	134
4	Meta analysis	272	14	Dysfunction	127
5	Rating scale	261	15	Mania	101
6	Impairment	255	16	Executive function	93
7	Euthymic patients	222	17	Memory	88
8	Deficits	202	18	Major depressive disorder	88
9	Depression	207	19	Neurocognition	79
10	Cognitive impairment	197	20	Association	71

**Fig. 7:**
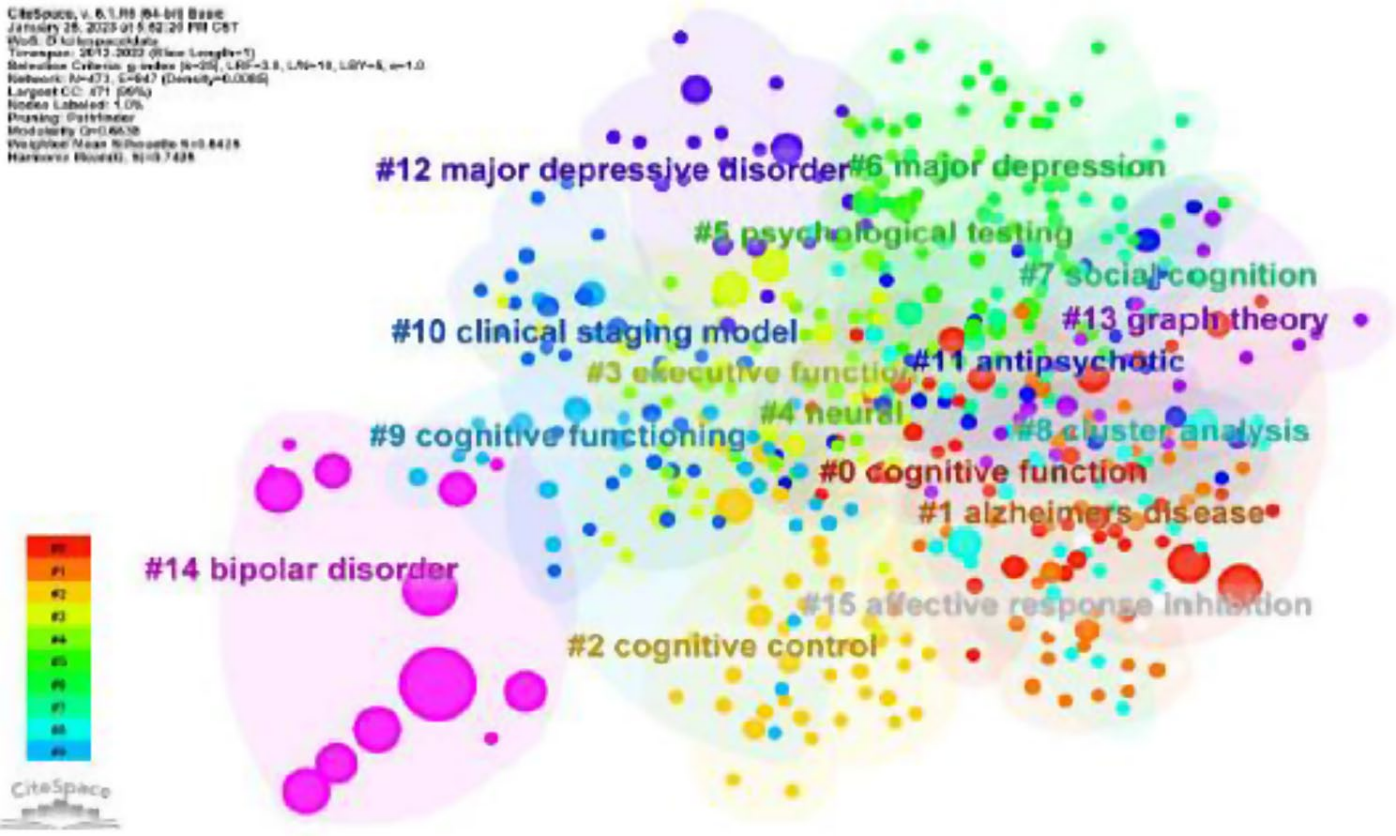
The cluster map of keywords

In CiteSpace, the LLR method was used to cluster the keywords (N = 473, E = 947) (Fig. 7, in supplementary material). All keywords were divided into 15 clusters with co-citation relationship, including the largest cluster “cognitive function” (#0), followed by “Alzheimer disease” (#1), “cognitive control” (#2) and “executive function” (#3). Clustering results point to clinical symptoms (including #0 cognitive function, #1 Alzheimer disease, #2 cognitive control, #3 executive function, #15 affective response inhibition) and diagnostic and intervention strategies (#5 psychological testing, #10 clinical staging model, #11 antipsychotic).

Generally, Q = 0.6638 (Q > 0.3) means that the clustering structure is significant. Silhouette: S value is the average contour value of clustering, it is generally believed that S > 0.5 clustering is reasonable, and S = 0.8425 (S > 0.7) means clustering is convincing (Fig. 7).

### Analysis of reference co-citation

**Fig. 8:**
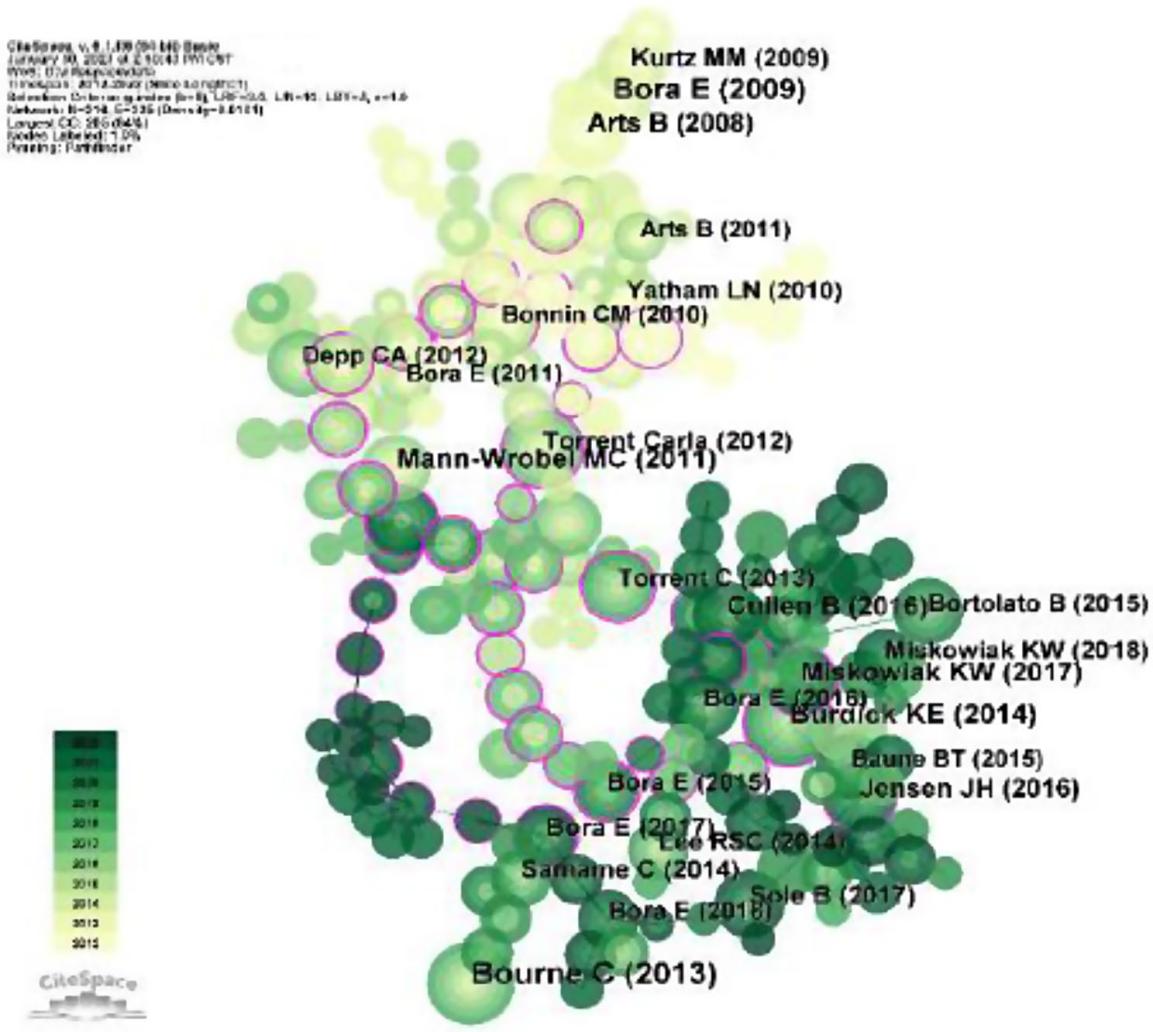
Co-occurrence analysis of references. (**A**) The nodes represent references. (**B**) The lines represent the relationships between the references and the common references of the collected studies

The CiteSpace software analysis was used to analyze studies published between 2012 and 2022, with 1-year time slice for analysis. The network diagram of the document is shown in Fig. 8, which consists of 216 nodes and 235 connections. The more times a document is cited, the greater the circle of the node. A circle with a purple outer ring indicates the document of intermediary centrality, meaning it is cited by several documents simultaneously. The color of the circle corresponds directly to the time slice, with yellow representing the earlier year and green representing more recent years. For example, light yellow represents co-cited studies in 2012, while dark green represents more recent co-references.

Table 5 shows the top 10 references, the most frequently cited is “Bourne C (2013)”, and the title of the article is “Neuropsychological testing of cognitive impairment in euthymic bipolar disorder: an individual patient data meta-analysis”, with 104 citations. This article is a meta-analysis describing reliable measures of cognitive impairment in bipolar patients: VLT, Digit Span, and TMT. Among the top ten cited articles, the highest centrality is “Bora E (2017)”, with the title “Meta-analysis of longitudinal studies of cognition in bipolar disorder: comparison with healthy controls and schizophrenia”, with a centrality of 0.22, which indicates that the article has important reference value in cognitive research of bipolar disorder.

**Table 5:** Top 10 most co-occurrence of references

Rank	Freq	Centrality	Label	Author	Year	Source
1	104	0.16	Bourne C(2013)	Bourne C	2013	ACTA PSYCHIAT SCAND
2	76	0.01	Bora E(2009)	Bora E	2009	J AFFECT DISORDERS
3	62	0.19	Burdick KE(2014)	Burdick KE	2014	PSYCHOL MED
4	60	0.08	Mann-Wrobel MC(2011)	Mann-Wrobel MC	2011	BIPOLAR DISORD
5	52	0.07	Miskowiak KW(2017)	Miskowiak KW	2017	BIPOLAR DISORD
6	50	0.09	Cullen B(2016)	Cullen B	2016	J AFFECT DISORDERS
7	46	0.02	Arts B(2008)	Arts B	2008	PSYCHOL MED
8	45	0.15	Jensen JH(2016)	Jensen JH	2016	J AFFECT DISORDERS
9	44	0.22	Bora E(2017)	Bora E	2017	PSYCHOL MED
10	44	0.11	Torrent Carla(2012)	Torrent Carla	2012	J CLIN PSYCHIATRY

### Burst analysis of keywords

**Fig. 9:**
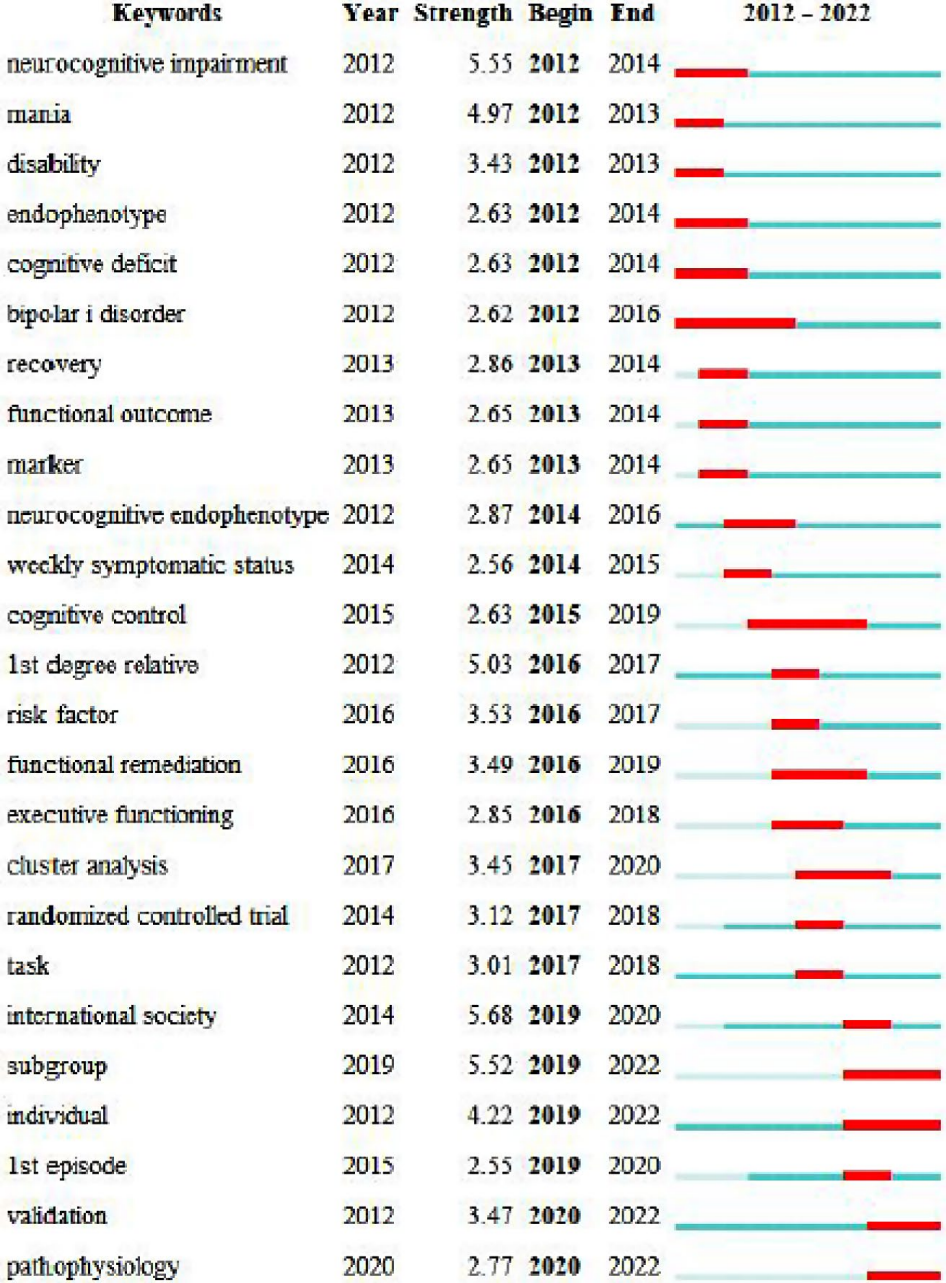
Top 25 Keyword with the strongest citation. The blue and white squares in each row on the right side of the figure correspond to the year of hotspot. Red squares represent the year of hotspot, and blue squares represent non-hotspot year. The recent successive red squares represent the research hotspots in recent wears

Burst words are the keywords that are frequently quoted and regarded as frontier topic indicators in a certain period. Figure 9 shows the top 25 keywords which are the most burst between 2012 and 2022. The most recent keywords are “pathophysiology”, “subgroup”, “individual”, “cognitive control” and “cluster analysis”. The keyword “neurocognitive impairment”, shows the reference burst to 5.55 began in 2012.

As can be seen from the figure, the research hotspots can be divided into the following three stages. The first stage was from 2012 to 2016. Focused on exploring cognitive impairment in patients with bipolar disorder, with keywords such as “neurocognitive impairment”, “cognitive impairment”, and“internal phenotype”,. The second stage was from 2016 to 2019, the keywords include “cognitive control”, “executive function”, “task”, and “risk factors”. This stage focused on investigating specific cognitive domains affected in bipolar disorder, utilizing cognitive task procedures and further integrating cognition with neurology. In the third stage, from 2019 to 2022, the keywords with bursts include “subgroup”、 “individual”、 “validation”、 “pathophysiology”. This stage emphasized the study of the neurocognitive subgroup within bipolar disorder, validating the scale for measuring cognitive function, and combining with pathophysiology. These studies provide practical evidence for the treatment of bipolar disorder.

## Discussion

This is the first bibliometric analysis of cognitive function in bipolar disorder. Our investigation covered 989 publications from WoSCC, most of them are original articles, and a few are review articles. The results show that during 2012–2022, the number of related publications increased significantly.

From the perspective of the authors. Vieta E, McIntyre RS, Vinberg M, and Miskowiak KW have published 20 or more articles. The articles published by Professor Eduard focus on cognitive dysfunction of schizophrenia and bipolar disorder. One of these articles points out that there is a widespread cognitive impairment in the first episode of mania, and its severity is lower than that of individuals with schizophrenia in their first episode. BD patients performed better than schizophrenia patients in verbal working memory, mental speed, executive control, and immediate verbal memory [11]. Both diseases have cognitive impairments, albeit with varying degrees of severity. Exploring the comorbidities between the two can lead to the development of more treatment options. In McIntyre RS’s articles, two of them examined the impact of obesity on the cognitive function of BD patients. The result shows that overweight/obese BD patients have significant cognitive defects and experience more severe cognitive impairment than normal-weight BD patients [12, 13]. Approved for obesity treatment, Liraglutide, a GLP-1R agonist, has shown promise in enhancing objective measures of cognitive function in adults diagnosed with mood disorders. This research suggests that Liraglutide could potentially serve as a therapeutic intervention to improve cognitive function in individuals with mood disorders [14]. This research finding highlights the possibility of investigating factors like obesity, which contribute to cognitive abnormalities in patients, as potential avenues for developing interventions to enhance cognitive function.

According to the analysis of centrality and author’s cooperative relationship, it can be known that Miskowiak KW cooperated with two productive authors, Vieta E and Vinberg M. The five articles, produced in collaboration with Vieta E, are systematic reviews or meta-analyses. Two of them focus on cognitive dysfunction in patients with bipolar disorder across different age groups. Euthymic youths with BD exhibit significant cognitive dysfunction in verbal learning and memory, working memory, visual learning, and memory domains. Further research has shown that euthymic adults with BD have more widespread cognitive impairment [15]. Euthymic older adults with BD have important deficits in almost all cognitive domains, particularly in the memory domain [16]. The impact of the disease varies among different age groups, with cognitive decline typically occurring as individuals grow older, resulting in irreversible damage. Treatment options can only enhance cognitive performance in patients to a certain extent. One article explores the effects of various medications on cognitive function of BD patients. The efficacy of N-acetyl cysteine, pregnenolone, ketamine, and pramipexole did not demonstrate any cognitive benefits, while mifepristone, galantamine, and insulin were shown to improve different areas of cognition [17]. In addition, they also conducted a functional magnetic resonance imaging study on individuals with BD, and the results showed that the neural basis of cognitive impairment in BD patients was a failure to recruit key regions in the CCN and to suppress task-irrelevant DMN activity during cognitive performance [18]. Miskowiak KW has published eight articles with Vinberg M, one of which is meta-analysis. From the perspective of the ‘cold’ and ‘hot’ cognition and neuroimaging, they found that the most promising specific endophenotypic marker for BD is the abnormalities within ‘hot’ cognition, which is represented by impairments in emotion processing and regulation and reward processing [19]. The remaining seven articles are experimental studies. There are four articles related to cognitive remediation. They first conducted erythropoietin (EPO) trials, which showed that EPO effectively promotes cognitive recovery in patients [20]. Then based on the above research, they conducted a study on Action-Based Cognitive Remediation (ABCR). The result showed that the ABCR can improve executive function and subjective cognitive functioning in BD patients [21, 22]. In another study, cognitive remediation (CR) was shown to improve subjective sharpness/mental acuity, verbal fluency and psychological quality of life in BD patients [23].

Most of the research on cognitive functioning in bipolar disorder was published in *Journal of Affective Disorders* (IF = 6.533, Q1), indicating it is currently the most popular journal in this research field. Among these journals, the journal with the highest impact factor is *Psychiatry Research* (IF = 11.225, Q1), followed by *Psychological Medicine* (IF = 10.592, Q1). Besides, *PLOS ONE*, which ranks 10th in terms of the number of published articles, is an open-source journal. This indicates that the presence of open-source journals has also promoted the development of this field, and it can obtain full-text documents for free, which facilitates knowledge dissemination and allows researchers to stay updated with the latest findings in the field of cognitive function in bipolar disorder. Through the co-citation analysis of journals, it can be found that the top cited journals are Q1 journals, which provide support for the study of cognitive function in bipolar disorder. More importantly, the most of research on cognitive function in bipolar disorder is published in clinical journals, which indicates that current research is in the clinical research stage.

In this bibliometric analysis, the majority of the related articles were published by authors from the United States, China, Canada, Spain, and Britain. According to the centrality, the United States and Britain had more cooperation with other countries, indicating their higher level of international cooperation. Although the number of articles published by China ranks second, showed that it had made extensive development in this field. However, due to a lack of collaboration with other productive countries, its influence is relatively low. Therefore, more international cooperation is needed in China. Spain ranks third in the number of articles published and had a significant presence due to the most active scholar, “Vieta E”, from the University of Barcelona. Vieta E set up a group to study bipolar disorder and depression, through experimental design and clinical trials, research on medication treatments, psychological therapy, and biophysical therapy, investigate the clinical development and progression of these disorders.

Keywords can be regarded as the core content of a specific article. Thus, keyword frequencies provide important clues about major trends in a research area [24]. Through the co-occurrence analysis of keywords, it can be found that apart from bipolar disorder and cognition, schizophrenia is also a high-frequency cited keyword, which indicates that one direction of research in this field is a comparative study of schizophrenia and bipolar disorder. This study compares the evolution of cognitive functioning in the same intervention mode and explores the fields of cognitive impairment. The research has shown that people with schizophrenia also perform significantly worse than people with bipolar disorder on social cognitive tasks such as theory of mind (ToM) and emotion recognition [25]. The keyword “Rating Scale” is also frequently cited, reflecting that a research topic in this research field is the analysis and measurement of cognitive scale for bipolar disorder, such as evaluating the degree of cognitive impairment in patients with bipolar disorder using different scales. Through cluster analysis, it can be found that the research mainly focuses on the cognitive impairments, diagnosis, and treatment of bipolar affective disorder in clinical settings.

Highly co-cited references are those that are frequently cited together by other articles, and thus, can be regarded as knowledge bases in a particular field [26]. In this article, the top ten cited literature are listed. The total number of citations can be regarded as an important indicator of interest in a specific research field. Nine of the articles in the top 10 co-cited references are meta-analyses. The tenth article is an empirical study published by Torrent Carla in 2012. This study points out the changes in cognitive impairment in BD patients during a longitudinal study: except for a worsening of executive function and slight improvement of attention, other cognitive fields remained stable [27]. The most frequently cited documents have been mentioned before and will not be explained here. The second most cited article is a meta-analysis published by Bora E in 2009. The results of this analysis show that response inhibition, set- shifting, verbal memory, and target detection impairments are potential candidate endophenotypes for BD. Euthymic patients may be associated with the medication they are taking and can also be influenced by disease-related factors [28]. The top 10 co-cited references focus on the following topics: meta-analysis, cognitive impairment, endophenotype, memory, executive function, neuropsychological test, and neurocognition. These are the research bases of cognitive function in bipolar disorder.

The burst detection analysis can show the interests of the research field and the changes in research hotspots over time. If a keyword is a high-frequency cited burst word, it indicates that the keyword has been actively discussed or used in a certain period [29]. From the citation bursts, we can conclude that the research on cognitive function in bipolar disorder mainly focuses on the following aspects: subgroup, individual, validation, and pathophysiology.

“subgroup”. There are three neurocognitive subgroups of BD. The “cognitively intact group” does not differ from HCs. The “Selective cognitive impairment group” has a lower cognitive score compared to HCs and one or two cognitive fields are damaged. The “Global cognitive impairment group” shows overall cognitive impairment [30–32]. There are differences in cognitive performance between subgroups of BD. BD-I performs significantly worse than BD-II in some cognitive performance, such as verbal memory, and processing speed [33]. However, BD-II has larger impairments in inhibition [34]. The study of cognitive subgroups and the varying impairments of the cognitive function in different subtypes of bipolar disorder will emerge as a prominent research focus in this field.

“individual”. Bipolar patients demonstrate increased intra-individual variability in cognitive processing, which can be observed through dispersion across tests within a single testing session or relative variability compared to their average performance over time [35, 36]. In the future, we can study the intra-individual variability of cognitive ability of BD patients and its clinical significance。.

“validation”. The related content is the validation of cognitive assessment for bipolar disorder. The effectiveness of a cognitive assessment tool may vary among different individuals or regions, so it is necessary to verify its validity when applied in new situations. ICAT, an internet-based cognitive assessment tool, has been found to be feasible for large-scale assessment and monitoring of cognition in the clinical management of bipolar disorder [37]. The reliability and validity of the new cognitive assessment tools may be verified in future research. By utilizing existing cognitive assessment tools, we can determine the extent of cognitive impairment in BD patients, which can help identify therapeutic targets. For instance, a study has shown that many patients with BD have sleep problems, which can impact the process of cognitive testing and the accuracy of test results. It indicates that sleep maybe a potential target for treating cognitive disturbances in BD [38].

“pathophysiology”, major depressive disorder (MDD) and BD exhibit similar microstructural abnormalities in anterior callosal fibers, which can be considered as a basis for the neuropathy physiology of these two disorder [39]. Additionally, some evidence indicates that there is a connection between the dysfunction of the the hypothalamic-pituitary-adrenal (HPA) axis and impairments in neurocognitive function in BD. Dopamine neurotransmission is also believed to play an important role in the pathophysiology of BD [40]. By incorporating these findings into a coherent pathophysiological model, future research can generate testable hypotheses.

## Strengths and limitations

Firstly, this study analyzes the research on the cognitive function of bipolar disorder using a bibliometrics system for the first time, providing valuable guidance for scholars interested in related research. Secondly, this article uses two bibliometrics tools, VOSviewer and CiteSpace, which have been widely used in the field of bibliometrics, ensuring an objective data analysis process.

At the same time, there are certain limitations. The data of this study only comes from the WoSCC database, other databases are ignored, and language restriction to English may result in the omission of relevant studies. Additionally, the time span considered in this study is from 2012 to 2022, excluding the year 2023 due to insufficient data.

## Conclusion

Cognitive function has important research value and promising prospects for application in bipolar disorder. In general, the number of research papers is increasing, and there is a need for stronger collaboration among countries. Each journal has its scope, and researchers can choose an appropriate journal based on their article. On the one hand, the study of the neurocognitive subgroup of bipolar disorder helps us determine cognitive domains that are impaired in individuals with the disorder. This knowledge is valuable for diagnosing the disease process of bipolar disorder. On the other hand, with the development of society, the cognitive function assessment tools of bipolar disorder are also constantly updated, and these tools can help us understand the general situation of the patient before formal treatment. Therefore, it is of great application value to continuously verify the reliability and validity of new cognitive assessment tools. In addition, more treatment options can be explored in combination with other areas.

### Electronic supplementary material

Below is the link to the electronic supplementary material.

Supplementary Material 1

## Data Availability

No datasets were generated or analysed during the current study.
