# Supplementary material for: A bibliometric and visual analysis of cognitive function in bipolar disorder from 2012 to 2022
Source: Ann Gen Psychiatry. 2024 Apr 18;23:13. doi: 10.1186/s12991-024-00498-x (PMC11027271; doi:10.1186/s12991-024-00498-x)
Supplement: Supplementary file 1 — Supplementary Material 1 [file 12991_2024_498_MOESM1_ESM.docx]

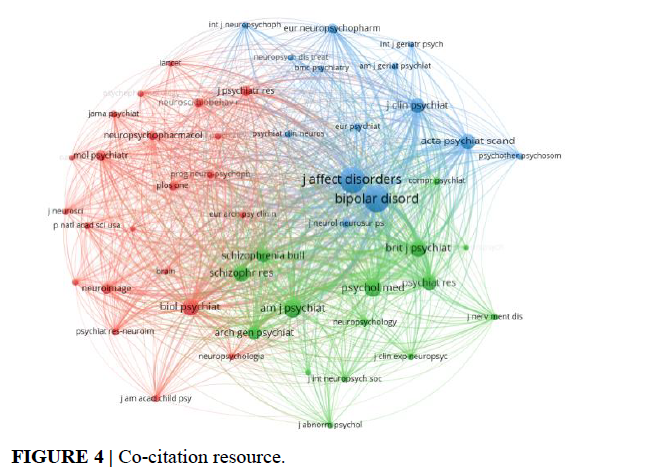


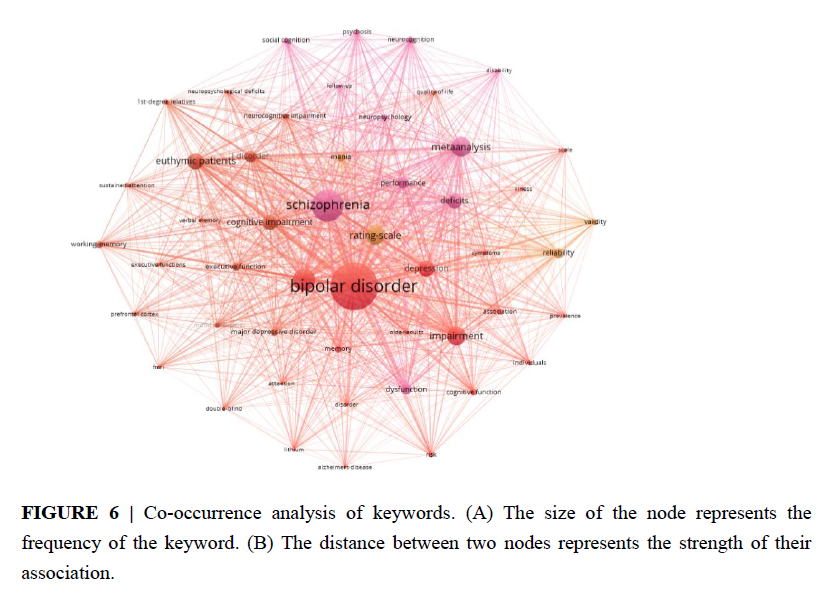


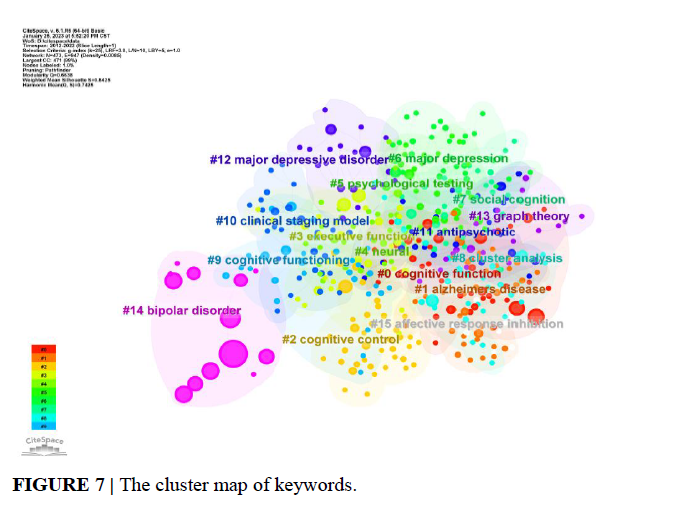


| Rank | Freq | Centrality | Label | Author | Year | Source |
| --- | --- | --- | --- | --- | --- | --- |
| 1 | 104 | 0.16 | Bourne C(2013) | Bourne C | 2013 | ACTA PSYCHIAT SCAND |
| 2 | 76 | 0.01 | Bora E(2009) | Bora E | 2009 | J AFFECT DISORDERS |
| 3 | 62 | 0.19 | Burdick KE(2014) | Burdick KE | 2014 | PSYCHOL MED |
| 4 | 60 | 0.08 | Mann-Wrobel MC(2011) | Mann-Wrobel MC | 2011 | BIPOLAR DISORD |
| 5 | 52 | 0.07 | Miskowiak KW(2017) | Miskowiak KW | 2017 | BIPOLAR DISORD |
| 6 | 50 | 0.09 | Cullen B(2016) | Cullen B | 2016 | J AFFECT DISORDERS |
| 7 | 46 | 0.02 | Arts B(2008) | Arts B | 2008 | PSYCHOL MED |
| 8 | 45 | 0.15 | Jensen JH(2016) | Jensen JH | 2016 | J AFFECT DISORDERS |
| 9 | 44 | 0.22 | Bora E(2017) | Bora E | 2017 | PSYCHOL MED |
| 10 | 44 | 0.11 | Torrent Carla(2012) | Torrent Carla | 2012 | J CLIN PSYCHIATRY |

**TABLE 5 |** Top 10 most co-occurrence of references.
